# Different doses of atorvastatin in the treatment of patients with cardiorenal syndrome Type-2: A retrospective cohort study

**DOI:** 10.12669/pjms.40.4.8706

**Published:** 2024

**Authors:** Ermiao Zhang, Tao Xu, Bin Zhang, Lei Meng

**Affiliations:** 1 Ermiao Zhang, Department of Coronary Herat Disease Intensive Care Unit, First Affiliated Hospital of Hebei North University, Zhangjiakou 075000, Hebei Province, P.R. China; 2 Tao Xu, Department of Coronary Herat Disease Intensive Care Unit, First Affiliated Hospital of Hebei North University, Zhangjiakou 075000, Hebei Province, P.R. China; 3 Bin Zhang, Department of Cardiology, First Affiliated Hospital of Hebei North University, Zhangjiakou 075000, Hebei Province, P.R. China; 4 Lei Meng, Department of Coronary Herat Disease Intensive Care Unit, First Affiliated Hospital of Hebei North University, Zhangjiakou 075000, Hebei Province, P.R. China

**Keywords:** Atorvastatin, Different doses, Cardiorenal Syndrome Type-2

## Abstract

**Objective:**

To assess the cardiorenal protective effects of different doses of atorvastatin in patients with cardiorenal syndrome (CRS) Type-2.

**Methods:**

Medical records of 113 patients with CRS Type-2, admitted to First Affiliated Hospital of Hebei North University from August 2021 to August 2022 and treated with atorvastatin, were retrospectively analyzed. Patients were retrospectively grouped based on the dosage of atorvastatin. A total of 38 patients who received 10mg/day atorvastatin were selected as a Low-dose group, 36 patients who received 20mg/day atorvastatin comprised a Medium-dose group, and 39 patients who received 40mg/day atorvastatin comprised a High-dose group. Cardiac function indicators (Left ventricular end-diastolic dimension [LVEDD], left ventricular end-stage systole diameter [LVESD], and left ventricular ejection fraction [LVEF]), renal function indicators (creatinine [SCr], serum uric acid [SUA], heme oxygenase-1 [HO-1], urinary albumin [UALB]), and inflammatory factors (Serum interleukin-6 [IL-6], hypersensitive C-reactive protein [hs-CRP], and tumor necrosis factor -α [TNF-α]) were compared between the three groups.

**Results:**

After the treatment, levels of renal and cardiac function indicators, and inflammatory factor indicators of the three groups were significantly improved compared to the before-treatment levels. The degree of improvement in the Medium-dose and the High-dose groups was significantly higher than in the Low-dose group (*p*<0.05). There were no significant differences in all cardiorenal function indicators and inflammatory factors between the Medium-dose and the High-dose groups after the treatment. During the treatment process, no adverse events were reported in all three groups.

**Conclusions:**

In the treatment of patients with CRS Type-2, medium dose (20mg/day) atorvastatin can have the same therapeutic effect as the high dose (40mg/day) treatment. Medium dose has a good protective effect on the heart and kidneys of the patients, and helps to reduce inflammatory reactions and improve heart and kidney function.

## INTRODUCTION

Cardiorenal syndrome (CRS) Type-2 mainly refers to chronic renal dysfunction caused by chronic heart failure.^1^,^2^ Cardiac and renal function are closely related through various dynamic and bidirectional mechanisms, and pathological changes in one organ can have adverse effects on the function of another distant organ.^2^,^3^ Several studies suggest that hemodynamics is the initiating factor for the onset of CRS Type-2.^4^,^5^ Hemodynamic disorders in chronic heart failure can activate neurohormonal mechanisms, promote renin release, induce myocardial fibrosis, worsen ventricular remodeling, and ultimately lead to cardiac failure, while inducing renal dysfunction.^3^–^5^

Multiple studies have shown that in addition to lowering blood lipids, statins also have the effect of protecting cardiovascular and renal systems through their anti-thrombotic, anti-inflammatory, and antioxidant effects, and the ability to promote the recovery of vascular endothelial function.^6^,^7^ At present, most studies have focused on the individual protective effects of statins on the kidney or heart, or on the protective effects of statins on the kidney or heart in the presence of renal and cardiac dysfunction. There is little research data on whether there is a significant difference in the protective effects of statins on the heart and kidney function and structure in patients with cardiorenal syndrome at the same time and at different doses.^8^ The aim of the current study was to explore anti-inflammatory and protective effects of atorvastatin, a commonly used drug for the treatment of cardiorenal syndrome, and to assess whether different doses of atorvastatin have differential protective effects on heart and kidneys of patients with CRS Type-2.

## METHODS

Medical records of 113 patients (56 males and 57 females) with CRS Type-2, treated in First Affiliated Hospital of Hebei North University from August 2021 to August 2022, were retrospectively analyzed. The ages of the patients ranged from 60 to 85 years, with an average age of 71.36±5.78 years. According to the treatment records, 38 patients received atorvastatin dose of 10mg/d and were assigned to the Low-dose group, 36 patients received a dose of 20mg/day and were assigned to the Medium-dose group, and 39 patients received a dose of 40mg/day and were assigned to the High-dose group.

### Inclusion criteria:

- Patients diagnosed with CRS Type-2.^9^
- The course of renal insufficiency is>3 months, and the course of cardiac insufficiency is>6 months.
- Age over 60 years old;

### Exclusion criteria:

- Patients who need long-term hemodialysis treatment;
- Accompanying malignant tumors;
- Patients with acute myocardial infarction or unstable angina pectoris and those with a recent history of cardiac surgery;
- Patients with blood system diseases, severe liver and kidney diseases, acute and chronic infections, and mental illness;
- Patients who have used statins in the past month;
- Incomplete clinical data.

### Ethical approval

The experimental design of this study has been reviewed and approved by the Ethics Committee of the First Affiliated Hospital of Hebei North University (Approval number: W2023022; date: 2023-05-19).

### Atorvastatin

Beijing Jialin Pharmaceutical Co., Ltd., specification: 10 mg/tablet, approval number: H19990258; Continuous treatment for six months.

The following baseline data of patients and relevant indicators were collected before and six months after the treatment:

### Renal function indicators

Serum from 3-mL of peripheral blood was analyzed using the BK-1200 automatic biochemical analyzer (Jinan Laibao Medical Equipment Co., Ltd.) to detect serum creatinine (SCr), serum uric acid (SUA), heme oxygenase-1 (HO-1), and urinary albumin (UALB) levels.

### Cardiac function indicators

Left ventricular end-diastolic dimension (LVEDD), left ventricular end-stage systole diameter (LVESD), and left ventricular ejection fraction (LVEF) were measured using the HDI-5000 Doppler echocardiography (Philips, USA).

### Inflammatory factors

Serum interleukin-6 (IL-6), hypersensitive C-reactive protein (hs-CRP), and tumor necrosis factor -α (TNF-α) were measured using enzyme-linked immunosorbent assay. The above reagent kits were all purchased from Shanghai Enzymes Biotechnology Co., Ltd.

### Statistical analysis

All data analyses were conducted using SPSS25.0 and PRISM8.0 software. The measurement data were represented by mean ± standard deviation, the comparison of measurement data between three groups was conducted using analysis of variance, and the pairwise comparison between groups was conducted using SNK test. The comparison of data at different time points in the same group was conducted using repeated measurement data analysis of variance. The counting data were represented by the number of cases and compared using chi square test. *p*<0.05 indicated a statistical difference.

## RESULTS

A total of 113 patients met the inclusion criteria. There were no significant differences in baseline data such as gender, age, comorbidities, and smoking history between the three groups (*p*>0.05) (Table-I).

**Table-I:** Comparison of the basic data between the groups.

Group	n	Gender (Male/Female)	Age (years)	Complications (Dyslipidemia/Hypertension)	Smoking history (yes/no)
Low-does group	38	19/19	70.42±5.74	10/14	17/21
Medium-dose group	36	20/16	71.36±5.90	11/9	16/20
High-dose group	39	17/22	72.28±5.70	13/14	13/26
*χ^2^/F*		1.077	0.998	2.235	1.343
*p*		0.584	0.372	0.693	0.511

**Fig.1:**
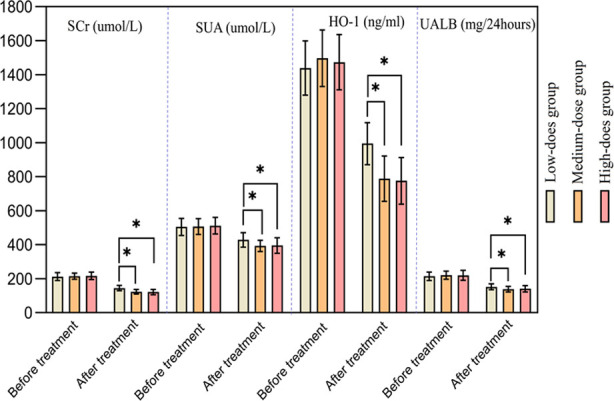
Comparison of renal function indicators between the three groups.

There was no significant difference in the renal function indicators among the three groups before the treatment (*p*>0.05). After the treatment, renal function indicators in all three groups decreased, and were significantly lower in the Medium-dose group and High-dose group compared to the Low-dose group (*p*<0.05). After the treatment, there was no significant difference in renal function indicators between the Medium-dose group and the High-dose group (*p*>0.05) Fig.1.

**Fig.2:**
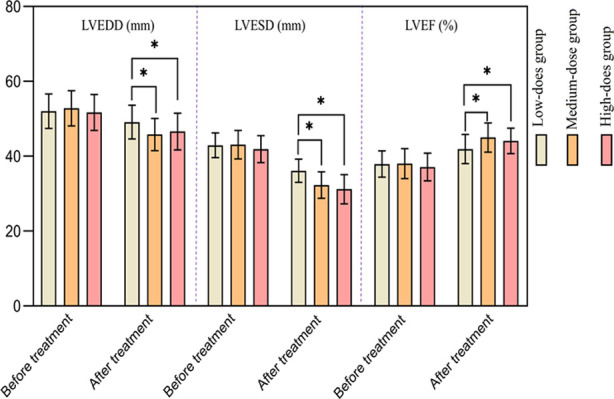
Comparison of cardiac function indicators between the three groups.

There was no significant difference in cardiac function indicators among the three groups before the treatment (*p*>0.05). After the treatment, the LVEDD and LVESD of the three groups decreased, while LVEF increased. After the treatment, LVEDD and LVESD of the Medium-dose and the High-dose groups were significantly lower, and LVEF was significantly higher than those of the Low-dose group (*p*<0.05). There was no significant difference in post-treatment LVEDD, LVESD, and LVEF between the Medium-dose and the High-dose groups (*p*>0.05) Fig.2.

**Fig.3:**
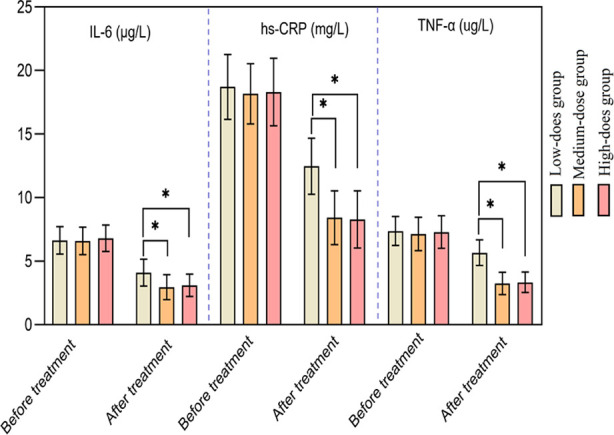
Comparison of inflammatory factor indicators between the three groups.a

There was no significant difference in the levels of various inflammatory factors among the three groups before the treatment (*p*>0.05). After the treatment, all inflammatory factor indicators in the three groups decreased, and were significantly lower in the Medium-dose and the High-dose groups compared to the Low-dose group (*p*<0.05). Levels of inflammatory indicators after the treatment were similar in the Medium-dose and the High-dose group (*p*>0.05) Fig.3. No adverse events were reported during the treatment in all three groups.

## DISCUSSION

The results of this study indicate that moderate doses (20mg/day) of atorvastatin can exert the same cardiorenal protective effect as high doses in terms of improving patients’ cardiac and renal function.

Atorvastatin, a 3-hydroxy-3-methylglutaryl CoA reductase inhibitor, can protect ischemic myocardium, inhibit cardiac remodeling, promote the recovery of cardiac autonomic nervous function, and thus protect cardiac function by downregulating the angiotensin-II (AngII) receptor.^10^,^11^ Recent studies have shown that atorvastatin not only improves the prognosis of cardiovascular disease patients and reduces the incidence of cardiovascular disease through its lipid-lowering effect, but also plays an antithrombotic, antioxidant, anti-inflammatory role. It helps to improve vascular endothelial cell function, eliminate free radicals, inhibit their production, as well as helps to inhibit the activation of the neuroendocrine system, thereby reducing kidney damage and improving kidney function.^12^,^13^

RCT by Yao Jianhua et al.^14^ showed that the addition of atorvastatin to the conventional treatment for cardiorenal syndrome can significantly improve renal function, and that the 40mg/day dose is more effective than the 20mg/day dose. Vogt L et al^8^ analyzed six double-blind randomized controlled trials and found that atorvastatin has a dose-dependent beneficial effect on renal function and related cardiovascular outcomes, with a dose effect of 80mg/day significantly higher than 10mg/day. However, considering the safety of the drug, the highest dosage of atorvastatin in previous treatments was 40mg/day. Chan JC et al^15^ conducted a safety assessment that included 2519 Asian patients receiving atorvastatin treatment, and demonstrated a lower incidence of adverse events/serious adverse events in Asian patients at doses of 10-40mg/day. Among the 113 patients included in this study, no adverse events were reported due to the use of atorvastatin.

Numerous studies have found that atorvastatin mainly improves renal blood flow and protects renal function by reducing the synthesis of heme oxygenase-I (HO-I) and enhancing the activity of nitric oxide.^16^,^17^ The results of our study showed that while all inflammatory factor indicators decreased after the treatment in all three groups, the moderate (20mg/day) and high (40mg/day) doses were associated with significantly lower levels of inflammatory factors than the low (10mg/day) dose (p<0.05). There was no significant difference in the levels of inflammatory factor indicators in patients in the Medium-dose and the High-dose groups (p>0.05). This indicates that moderate doses of atorvastatin can exert the same anti-inflammatory effects as high doses. Liu L et al.^12^ found that compared with patients who received 20mg atorvastatin, patients who were treated with 40mg atorvastatin had lower levels of TNF-α and hs-CRP, but higher levels of NO, and that higher dose of atorvastatin could regulate the levels of inflammatory factors in patients, which is consistent with the results of this study. We may speculate that this effect is due to ability of atorvastatin to reduce the concentration of intracellular calcium ions, inhibit Na^+^ and Ca_2_^+^ exchange, and promote NO decomposition in endothelial cells, thus better protecting myocardial cells.^18^,^19^ At the same time, statins can also reduce the tension of fiber caps, reduce the lipid nuclei in plaques, and thus suppress inflammatory reactions.^19^

### Limitations

The main limitation of this study is its retrospective single-center nature with a small sample size, which may result in a certain patient selection bias. Further multicenter, large-scale prospective controlled studies are needed to confirm the conclusions of this study and to assess whether a higher dosage (80mg/day) can better improve the patient’s cardiac and renal function. Furthermore, different patient groups need to be selected to observe the effect of atorvastatin on neurological function.

## CONCLUSION

In the treatment of patients with CRS Type-2, medium dose (20mg/day) of atorvastatin can have the same therapeutic effect as the high dose (40mg/day), and has a good protective effect on the heart and kidney, helping to reduce inflammatory reactions and improve heart and kidney function of patients with CRS Type-2.

### Authors’ contributions:

**EZ** conceived and designed the study.

**TX, BZ and LM** collected the data and performed the analysis.

**EZ** was involved in the writing of the manuscript and is responsible for the integrity of the study.

All authors have read and approved the final manuscript.
